# In vitro assessment of benzothiadiazole-based photoactive polymers against ovarian, prostate and bladder cancer cell lines for photodynamic therapy

**DOI:** 10.1007/s12672-025-03654-1

**Published:** 2025-10-14

**Authors:** Rolan Mansour, Fawziye Tarhini, Nicolas Bruce, Jamie Blanche, Mahmoud Wagih, Ahmad Taha, Karin Williams, Jonathan Copper, Muhammad Imran, Karin Oien, Hadi Heidari, David Flynn

**Affiliations:** 1 James Watt School of Engineering, University of Glasgow, Glasgow, G12 8QQ UK; 2 School of Cancer Sciences, University of Glasgow, Glasgow, G61 1QH UK

**Keywords:** Photodynamic therapy (PDT), Microsystem, Biomedical technology, Materials, Cancer therapy, Photosensitizers

## Abstract

There are unmet clinical needs of bladder cancer associated with its late detection, limited treatment options, and high mortality rate. In response to this, we present the results of the in-vitro assessment of photodynamic polymers, prior to their integration into a wireless biomedical implant for in-situ photodynamic therapy (PDT). PDT selectively activates light-sensitive photosensitizers, to kill cancer cells without the adverse effects associated with systemic therapies, such as chemotherapy. PDT offers significant advantages in hypoxic tumors, including oxygen-independent cytotoxicity known to impede the curative efficiency of radiotherapy. Accordingly, we report on the design methodology and microfabrication process of an in-situ PDT wireless microsystem as a novel treatment modality. We present the preliminary results from three benzothiadiazole-based polymers that are evaluated as solid support photosensitizers for the inhibition of cancer growth in three cell lines. Cell lines represent ovarian, prostate and bladder cancer, the reduction in cell number was assessed with Sulforhodamine B assays. Following Polyamide, aminophenyl- (PA-ABT) treatment, cells were subjected to a range of 420 nm light exposures and fluorimetry was used to determine cell growth. PA-ABT was shown to cause growth inhibition only at higher concentrations in all tested cell lines. PA-ABT can be considered as a promising candidate for in-situ PDT treatment due to its inhibitory effects in cancer cell lines open new possibilities for mono or combination light-based therapy in cancer treatment for previously inaccessible cancers.

**Supplementary Information:**

The online version contains supplementary material available at 10.1007/s12672-025-03654-1.

##  Introduction

The global impact of cancer has serious implications for the socioeconomic health of nations of all economic status, and at the individual patient level, the level of pain and suffering is considerable. Both the incidence and prevalence of cancer are rising globally. It is anticipated that based on population growth and aging, the global cancer burden will grow to 29.4 million cases annually in 2040 [1]. 

Treatments for cancers are largely based on the trilogy of surgery, chemotherapy and radiation and combinations thereof. For example, in the case of bladder cancer in the USA, 75% of patients initially present with non-muscle invasive bladder cancer, but further stratification reveals that only 50% are considered low risk non muscle invasive bladder cancer [2] highlighting the complexity of patient population selection for new or alternate therapies. Photodynamic Therapy (PDT) has generally been considered a ‘niche’ treatment for the treatment of skin conditions but its use in accessible sites such as lung and bladder has been exploited [3, 4]and advances in light delivery should enable treatment of tumours deep within the body. PDT was approved by the FDA as the first drug-device combination treatment, almost two decades ago, however, it is clinically underestimated and underutilized. Although PDT is a newly emerging treatment modality, it is already proven to be a successful and clinically approved therapeutic approach that is specifically used for the treatment and management of neoplastic and non-malignant types of cancers such as skin cancer.

##  State of the art

PDT is minimally invasive treatment modality that kills cancer cells by selectively activating cytotoxic medicines known as photosenzitisers (PSs) optically with the help of a light source [5]. PDT involves the injecting of PSs, resulting in the generation of reactive oxygen species when absorbing enough light at the correct wavelength when concentrated in the tissue, which can destroy tumour tissue. PDT is expected to grow further as research advances, both as a stand-alone treatment and in conjunction with other therapies [6]. 

**Fig. 1:**
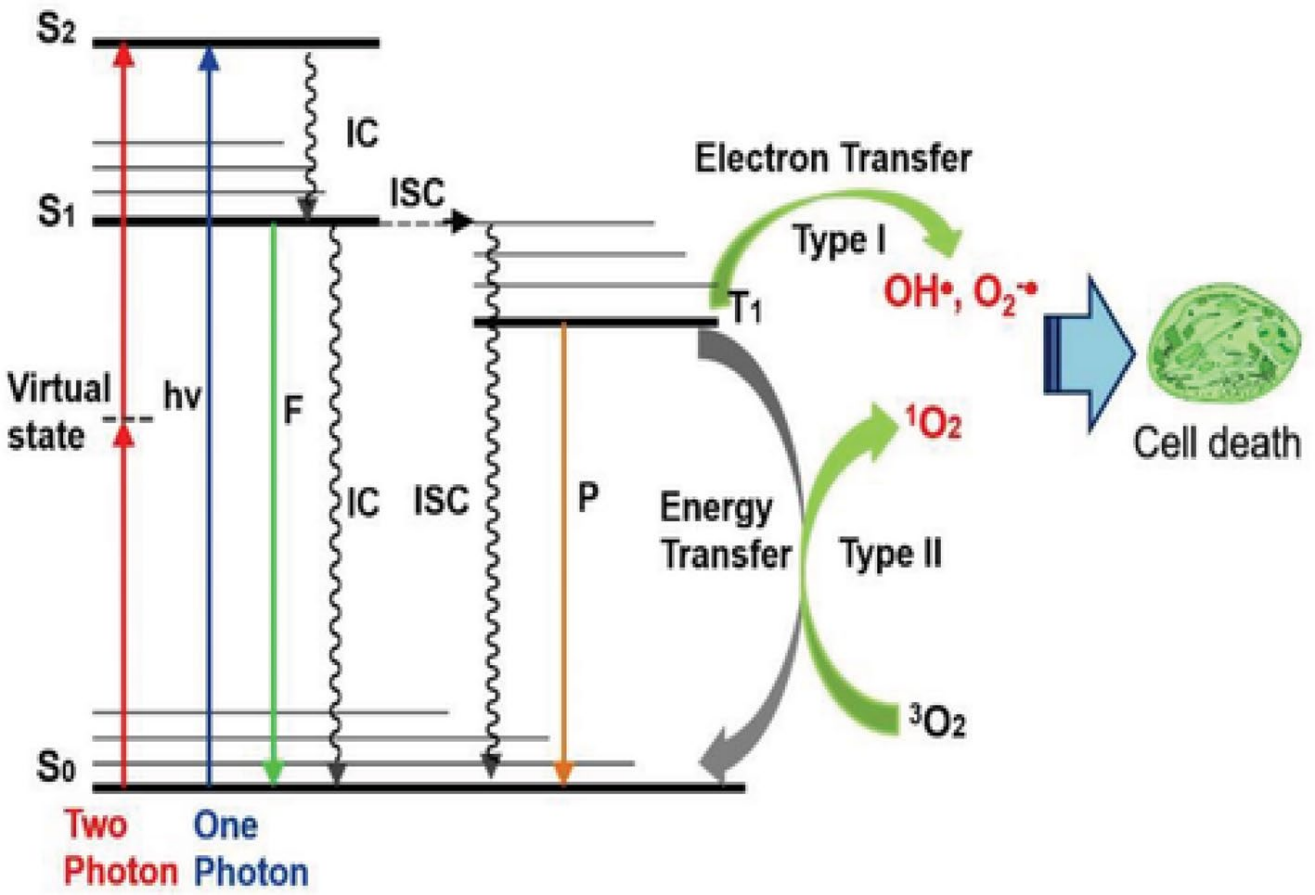
Graphical illustration of the photophysical and photochemical mechanisms of type 1 & type 2 PDT [10].

Spatial control is provided by the synergistic combination of two otherwise non-toxic elements: light and the PS. Only when exposed to a particular wavelength of light the PS can create cytotoxic agents. Upon photon absorption, the PS changes from the ground singlet state (S0) to an excited singlet state (S1). When the molecule relaxes back to its ground state, it may experience Type I and Type II reactions [7, 8] These are photochemical reactions that involve the production of highly reactive form of molecular oxygen (O_2_) that has absorbed energy known as singlet oxygen (^1^O_2_). Radicals are highly reactive species due to their unpaired electrons; these radicals can react with O_2_ molecule to produce singlet oxygenated products (^1^O_2_) through Type I reaction. In type I PDT reactions, the production of superoxide radicals and electron transfer mechanisms, are less dependent on oxygen. In Type II reaction, ^1^O_2_ is produced through the direct energy transfer to O_2_ molecule [9]. **(**Fig. 1**)**.

PDT has been successfully used in the treatment of several superficial and localized malignancies, including actinic keratosis, basal cell carcinoma, early-stage non-small cell lung cancer, and esophageal cancer [11]. However, its broader clinical application remains limited by key challenges, including the poor penetration depth of activating light, oxygen dependency, and limited efficacy in treating deep-seated or systemically disseminated tumors [12]. These limitations have motivated the development of advanced delivery systems—such as nanoparticle formulations, polymer-based carriers, and implantable light sources—to enhance tumor selectivity and improve PDT efficacy in more complex clinical contexts.

##  PA-ABT polymer a potential candidate for PDT

**Fig. 2:**
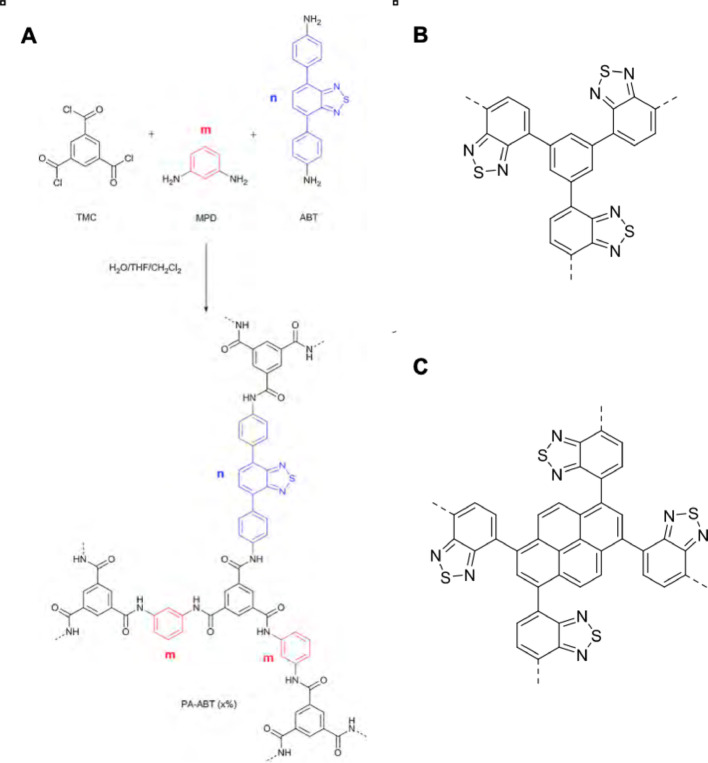
**A** Synthetic scheme for the photoactive polymer and representation of a possible fragment of the polymer network from Shen et al. (2016). PA-ABT was prepared via a metal-free polymerisation reaction between ABT, m phenylendiamine (MPD) and trimesoylchloride (TMC). ABT contains diamine groups similarly to MPD, allowing it to undergo polymerisation with TMC. Therefore, replacing MPD with select amounts of ABT allows incorporation of ABT into the polymer backbone. The molar ratio of (MPD + ABT) to TMC was fixed at 1.45. The final polymer was named PA-ABT (x%) where x% represents the molar percent of ABT in (MPD + ABT). **B** Monomeric structure of Benzothiadiazole-based conjugated polymer 1 (BTP-1). **C**Monomeric structure of Benzothiadiazole-based conjugated polymer 2 (BTP-2)

PDT and PS have a wide range of biological applications out with disease treatment. PS can be immobilised into solid supports such as chitosan and silica to prevent photobleaching in this high volume treatment modality [13]. This immobilization has applicability to PDT in patients as it can provide localised application of the PS potentially allowing for a single invasive application to the bladder that remains in contact with the lesion even after multiple urine voids. This permits an implantable light source to be used over a period of time without requirement for repeated PS application. In their recent study, Shen et al., 2016 have synthesized a novel conjugated photosensitizer known as PA-ABT polymer (ABT incorporated polyamide). The polymer can be synthesized in a simple process through a polymerization reaction. The photosensitizer monomer, an aminophenyl-substituted benzothiadiazole derivative (ABT) was inserted into a polyamide (PA) backbone to produce PA-ABT **(**Fig. 2A**).** The simple synthesis process of PA-ABT polymer that can be scaled up can be an advantage for the development of affordable and cost-effective treatments. Moreover, the structure of the polymer can be customised to optimise its qualities and properties for specific clinical requirements. For example, the polymer backbone can be changed to improve its performance, or the absorption wavelength can be adjusted or changed for better clinical performance, its incorporation did not reduce the ability of the photosensitizer to produce ^1^O_2_ [14]. These material properties make PA-ABT an effective candidate for PDT, enabling targeted, efficient, and controlled cancer therapy with minimal side effects [15]. PA-ABT and other benzothiadiazole-based polymers are a high singlet oxygen generator, and ^1^O_2_ is indeed the major ROS responsible for the cytotoxic effects in PDT. PA-ABT is mainly biocompatible, indicating a lower probability of inducing unfavourable immunological responses in the body, a vital characteristic for any therapeutic agent [16]. 

Additionally, with its chemical stability properties, PA-ABT polymer remains stable in the biological environment until it reaches its targeted tissue, where it can be activated by light. Moreover, PA-ABT polymers can be chemically altered to incorporate targeting ligands, providing a selective and specific target function properties of the polymer, which improve their ability to selectively accumulate in tumour [17]. PA-ABT polymer possess a minimal dark toxicity, where it shows low activity without light activation, which can be used in future for clinical applications with a greater safety [12]. Although ABT-incorporated polyamide polymers are insoluble, they may still be adapted for therapeutic use through formulation as nanoparticles or microparticles suspended in biocompatible liquids. These particles can encapsulate both ABT and a photosensitizer, allowing for administration via clinically established routes such as intravenous injection or local instillation—approaches commonly employed in PDT [12]. Intravenous injection and local administration (e.g., intravesical instillation for bladder cancer) remain the most clinically feasible delivery methods for nanoparticle-based PDT at this time [18]. Additional delivery strategies, including oral ingestion, implantable devices, surface coatings, or hydrogel composites, are currently being investigated in preclinical models [19, 20]. In such systems, PA-ABT can be incorporated into hydrogels or biodegradable carriers designed for topical or injectable applications. However, these alternative methods remain at an experimental stage, and further research is needed to assess their feasibility and safety in clinical settings. These systems allow for controlled or localized drug release without requiring the polymer to dissolve, making them effective for long-term or targeted treatment. In the case of PDT, the polymers can be directly delivered to the treatment site and activated by light exposure, enabling localized therapeutic action. Moreover, after irradiation, the PA-ABT polymer undergoes degradation triggered by ROS generated during PDT. The polymer contains ROS-sensitive linkages that break down upon light activation, leading to disassembly of the nanostructure and release of the active agent. The degraded fragments are designed to be biocompatible and small enough for renal clearance, ensuring safe elimination from the body.Therefore, PA-ABT can be a novel immobilized PS to be used within PDT to target cancer.

###  Bladder, ovarian and prostate cancer types are potential candidates for PDT

While early PDT studies often utilized a range of tumor models, including rodent systems, in this study we selected well-characterized human cancer cell lines (T24, PC-3, and SK-OV-3) to ensure experimental reproducibility and relevance to the clinical targets of interest, bladder, prostate, and ovarian cancer.

There are several biological, clinical and therapeutic reasons for using prostate, bladder and ovarian cancer cell lines for PDT. These cancer types are very relevant, possess consistent biological behavior, ease of in vitro handling, and exhibit characteristics that make them potential and suitable candidates for PDT modality. In the laboratory settings, these cancer types are highly available, widely used cell lines, and they have been well established which can be reproduced for in-vitro studies of PDT efficacy. These cell lines have been extensively used in many PDT studies, providing a basis for comparison and standardization in research.

Bladder cancer can be localized, thin and superficial if diagnosed at early stages, making PDT a novel treatment approach that can be effective compared to other treatment modalities. Recently, PDT is frequently used in clinical practices to treat superficial bladder cancer, where light can directly penetrate the bladder using cystoscopy, targeting directly the tumour lesion with minimal tissue damage to healthy surrounding tissue, providing a non-invasive, localized and pleasant treatment [21]. In this study we used T24 urinary bladder cell Line, which represents a transitional cell carcinoma that is considered the most common type of bladder cancer. This robust transfectable cell Line displays a fast proliferation rate and adherent properties and can be grown as 3D spheres. Furthermore, T24 cells have been used extensively to investigate uptake of several photosensitizers. Moreover, the study presented here correclty notes that the wavelength (420 nm) wavelength used in this study (420 nm) aligns with the optical properties of superficial early-stage bladder cancers (e.g., NMIBC), which are accessible and thin enough for effective light penetration.

Ovarian cancer even it is characterized at later stages, it is considered the leading cause of death among gynaecological cancers as this type of cancer characteristically invade the entire peritoneal cavity. Surgery or irradiation alone are not effective for treating ovarian cancer when it spreads, additionally, many types of ovarian cancers demonstrate high resistance to chemotherapy, stressing the need for novel and effective therapeutic approach. Although ovarian cancer is often diagnosed at an advanced stage, its high rate of locoregional recurrence and the need for targeted, less invasive therapies suggest that PDT may offer potential benefits, particularly for residual or recurrent disease in surgically accessible sites. PDT could potentially be used to limit the burden of peritoneal cavity disease. PDT offers a promising modality in treating ovarian cancer by targeting surgically accessible or confined metastatic lesions, such as peritoneal implants.The light can be delivered using laparoscopy, making it minimally invasive approach [22]. 

Prostate cancer represents a prime candidate for PDT particularly for its localized and recurrent characteristics and its varying tumour microenvironment. PDT in prostate cancer represents a minimally invasive treatment regime that could be offered as an alternative to watchful waiting or to provide additional augmentation to nerve sparing surgery with less side effects. PDT is explored in treating prostate cancer particularly at its early stages or when tumour is confined to the prostate. Moreover, the prostate a good candidate for PDT as it is accessible for light delivery using transrectal ultrasound [23]. Because the prostate is situated near the rectum, it is position makes it easily accessible for light delivery through the use of transrectal ultrasound. This is a technique commonly used in prostate imaging, where an ultrasound probe is inserted into the rectum to visualize the prostate. This allows for precise targeting of the prostate with light, ensuring that the PDT treatment is focused on the cancerous areas [24]. PC3 prostate cancer cells are a high-grade cancer representing the disease state that is considered terminal with conventional treatment.

Although the use of specific cell lines is not strictly required to demonstrate selective uptake, they provide a controlled system for assessing polymer interaction, photosensitizer localization, and photodynamic response, which collectively serve as a proxy for selective targeting of neoplastic tissue.

##  Aim of this study

The aim of this study is to explore whether PA-ABT and associated polymeric photosensitizers show efficacy when targeted against human cancer cell Lines in vitro. Furthermore, we assess whether the polymers can be administered in such a way that they are non-toxic in darkness whilst being efficacious on exposure to Light, a beneficial attribute for controlled, targeted therapy. Treating cell lines from 3 different tissues has shown promising results but also variability in sensitivity between cells. This suggests that as a possible PDT treatment, such polymers may only be effective in specific cancers.

Furthemore, in this study we introduced the use of a newly synthesized benzothiadiazole-based polymer (PA-ABT) as a light-activated agent with potential photodynamic activity. Our results demonstrate its selective cytotoxicity under light exposure in certain cancer cell lines, with minimal dark toxicity—highlighting a favorable therapeutic index. Additionally, we report unexpected photoprotective effects at lower concentrations, which warrant further mechanistic investigation.These findings provide preliminary yet promising data on the utility of PA-ABT in light-controlled cancer therapy, and we demonstrated how this contributes to the evolving field of PDT, particularly for localized and superficial tumors such as early-stage bladder cancer.

##  Methods

Three human cell lines were used (ovarian adenocarcinoma SK-OV-3, prostate adenocarcinoma PC-3 and bladder carcinoma T24/83). Cells were seeded at their appropriate densities and treated 48/72 hours following incubation with PA-ABT, BTP-1 or BTP-2 polymers then exposed to various 420 nm Light exposures. Following 4/5 days’ growth cells were stained and viability assessed by Sulforhodamine B assay.

###  Cell lines and culture

Well established and characterised cell lines were selected for these studies to represent three anatomical sites of interest for our PDT studies. Three cell lines were used: SK-OV-3 (Human Caucasian ovarian adenocarcinoma), PC-3 (Human Caucasian prostate adenocarcinoma) and T24/83 (Human bladder carcinoma) lines. All cell lines were purchased from the European collection of authenticated cell cultures (ECACC). Culture conditions were those recommended by the ECACC. SK-OV-3 cells were cultured in RPMI 1640 Medium (Thermo Fisher Scientific) supplemented with 10% fetal bovine serum (Thermo Fisher Scientific) and 1% penicillin-streptomycin (10,000 U ml-1) (Thermo Fisher Scientific).

PC-3 cells were cultured in Ham’s F12 (Coon’s Modification) nutrient mixture (Sigma-Aldrich) supplemented with 7% fetal bovine serum, 2mM glutamine (Thermo Fisher Scientific) and 1% penicillin-streptomycin. T24/83 cells were cultured in McCoy’s A5 Medium (Thermo Fisher Scientific) supplemented with 10% fetal bovine serum and 1% penicillin-streptomycin.

Cell Lines were split when sub-confluent 70–80% using 0.05% trypsin-EDTA (Thermo Fisher Scientific) and incubated in normoxia 21% O_2_ at 37 °C, 5% CO_2_.

###  Sulforhodamine B (SRB) assay

Cells were seeded at appropriate densities into 96-well plates (Thermo Fisher Scientific) with 200µL media/well; SK-OV-3–1 × 10^3^ cells/well, PC-3–4 × 10^3^ cells/well, T24/83–5 × 10^2^ cells/well. Plates were treated following 48/72 hours’ incubation. Wells were fixed following appropriate time courses by addition of 50µL 25% trichloroacetic acid (Sigma- Aldrich) and washed with H_2_O following 1 h at 4 °C. Plates were dried for 2–3 hours in an oven at 50 °C before addition of 50µL 0.4% sulforhodamine B (Sigma-Aldrich) (dissolved in 1% acetic acid) to each well. Following 30 min, plates were rinsed with 1% acetic acid and left to dry. Once dry, 150µL of 10mM Tris buffer solution (pH 10.5) (Sigma-Aldrich) was added to each well and plates were left on a rocker at room temperature for 1 h. Plates were then read at 540 nm on a Biohit BP800 microplate reader.

###  Treatment

Due to insolubility, polymer treatments were first suspended in appropriate media for the cell Lines at appropriate concentrations. Wells were aspirated and 200µL of the required treatment containing media added. Toluidine Blue O (Acros Organics) treatments were administered similarly. Treatments were given under standard lighting. To reduce light exposure, plates were covered with tinfoil between treatments and only left uncovered for short periods.

###  Light treatment

Plates requiring Light exposure were incubated for 10 min following media change of all plates in that experiment. As only one plate could be exposed at a time, plates were treated from the shortest to longest Light exposure time. Plates were placed in a 25× 15 cm polystyrene box with a 420 nm LED light source (OSA Opto - OLM-18D) suspended by a cut out in the lid ~ 13 cm above the plate. The inside of the box was at first plain white polystyrene but was later lined with black card. Plates were treated outside of tissue culture with a closed lid.

####  Calculation of the light dose

The information for calculating the light dose were obtained from the following link.

The total size of the Light source is 25 mm×25 mm = 6.25cm^2^ (light emitting area). In this study, *LED module OLM-018* UV400 Air mode LED Light source was used with typical power density of 1.9 W/cm^2^(the configuration of lamp at 1.9 W/cm^2^ to account for 1 h of illumination), then the total emitted Light by the machine is 1.9 W×6.25 = 11.877 W. Accordingly, at 13 cm of height (LED light source suspended by a cut out in the lid ~ 13 cm above the plate) 11.8 W is spread over an area of (25 cm×15 cm) = 375cm^2^ (polystyrene box), therefore, 11.8 W/375cm^2^ = 0.0315 W/cm^2^.

If 1 W = 1 J/second, then 0.0315 × 60 min×60c/per min = 113 J/cm^2^. Accordingly, the total Light dose is 113 J/cm^2^.

###  Statistical analysis

Data were analysed using one-way ANOVA and corrected by Tukey’s multiple comparisons test, statistical significance was defined as *P* < 0.05 throughout. Statistical analysis and graphing were performed in GraphPad Prism 6.

###  Polymers

Structure and synthesis of PA-ABT and structures of the BTP-1 and BTP-2 benzothiadiazole- based conjugated microporous polymers are displayed in **(**Fig. 4A, B and C). Polymers were provided by Dr Filipe Vilela, Heriot-Watt University, Edinburgh.

####  Characterisation of benzothiadiazole polymers

PA-ABT of molecular formula C_6_H_6_BrN_2_O_2_ and molecular weight of approximately 196.0 g/mol was prepared via a metal-free polymerisation reaction between ABT, m- phenylendiamine (MPD) and trimesoylchloride (TMC). ABT contains diamine groups like MPD, allowing it to undergo polymerisation with TMC. Therefore, replacing MPD with select amounts of ABT allows incorporation of ABT into the polymer backbone. The molar ratio of (MPD + ABT) to TMC was fixed at 1.45. The final polymer was named PA- ABT (x%) where x% represents the molar percent of ABT in (MPD + ABT) (Fig. 2A). Both BTP-1 and BTP-2 compounds share a similar structure C_18_H_18_N_4_O_4_S and molecular weight of approximately 370.43 g/mol.

###  UV-vis absorption and fluorescence spectra of ABT and PA-ABT (x%)

**Fig. 3:**
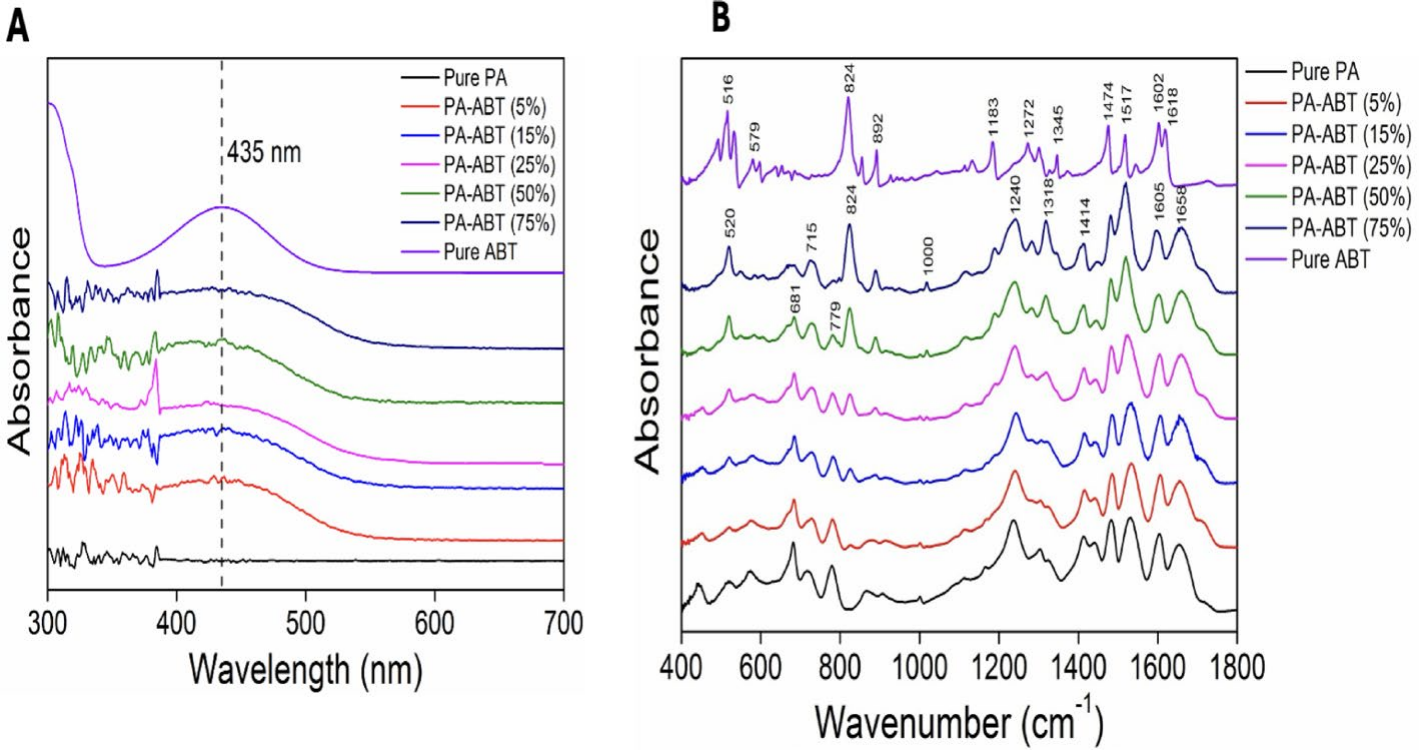
UV-vis absorption and fluorescence spectra of PA-ABT polymer. **A** UV/Vis spectra of pure ABT, PA-ABT (x%) and pure PA. **B** FT-IR spectra of pure ABT, PA-ABT (x%) and pure PA

The broad absorption band between 350 and 500 nm, with a maximum at 435 nm (Soret band), is representative of the UV/Vis absorption spectra **(**Fig. 3A**)** of ABT and is attributed to the transition from the electron-donating to the electron-accepting BT moiety. After ABT was added to polyamide, this absorption band slightly shifted, confirming that there are no electrical interactions between the TMC and ABT monomers. In contrast, there was no adsorption peak at 435 nm for the pure PA [14]. 

The FT-IR spectra of pure PA, pure ABT, and PA-ABT (x%) were shown in Fig. 3B. All samples showed peaks at 1470–1520 cm^–1^ and 1517–1618 cm^–1^, which were linked to the aromatic rings’ C = C stretching and the amide group’s N–H bending, respectively. The amide group’s C = O stretching was determined to be the cause of the peaks at 1658 cm-1 that were seen in PA and PA-ABT (x%) but not in ABT monomer. However, as they were seen for ABT and PA-ABT (x%) but not for pure PA, the peaks at 824 cm^−1^ and 892 cm^−1^ were the distinctive peaks for the BT moiety.

ABT monomer has been successfully incorporated into the PA backbone as evidenced by the rising intensity of the BT peaks with increasing ABT in PA-ABT (x%).

The rationale for selecting 420 nm Light when using PA-ABT photosensitizers in treating in our study is because of the photophysical properties of the PA-ABT photosensitiser, which can also be related to the biological effects of the generated ROS on the treated cells. PA-ABT has a strong absorption peak maximally around 420 nm **(**Fig. 3A**)** and this is mainly due to the presence of the conjugated aromatic molecule which forms a main part of PA-ABT structure **(**Fig. 2A**)**. At 420 nm Light wavelength, the photodynamic reaction can be activated in the presence of PA-ABT which triggers further biological effects and cellular damage due to the generated ROS. When PA-ABT absorbs the 420 nm light, it undergoes a transition process to an excited singlet state passing through the intersystem crossing phase and finally reaching the triplet state, where energy transfer to O₂ takes place to produce ¹O₂ and other ROS, which further can cause cytotoxic effects such as DNA damage and mitochondrial dysfunction. Moreover, the 420 nm wavelength is considered practical and precise for controlled in-vitro studies, while this wavelength is not ideal for deep seated tumours in-vivo, however, it is considered an excellent wavelength for surface and monolayer cell models.

**Fig. 4:**
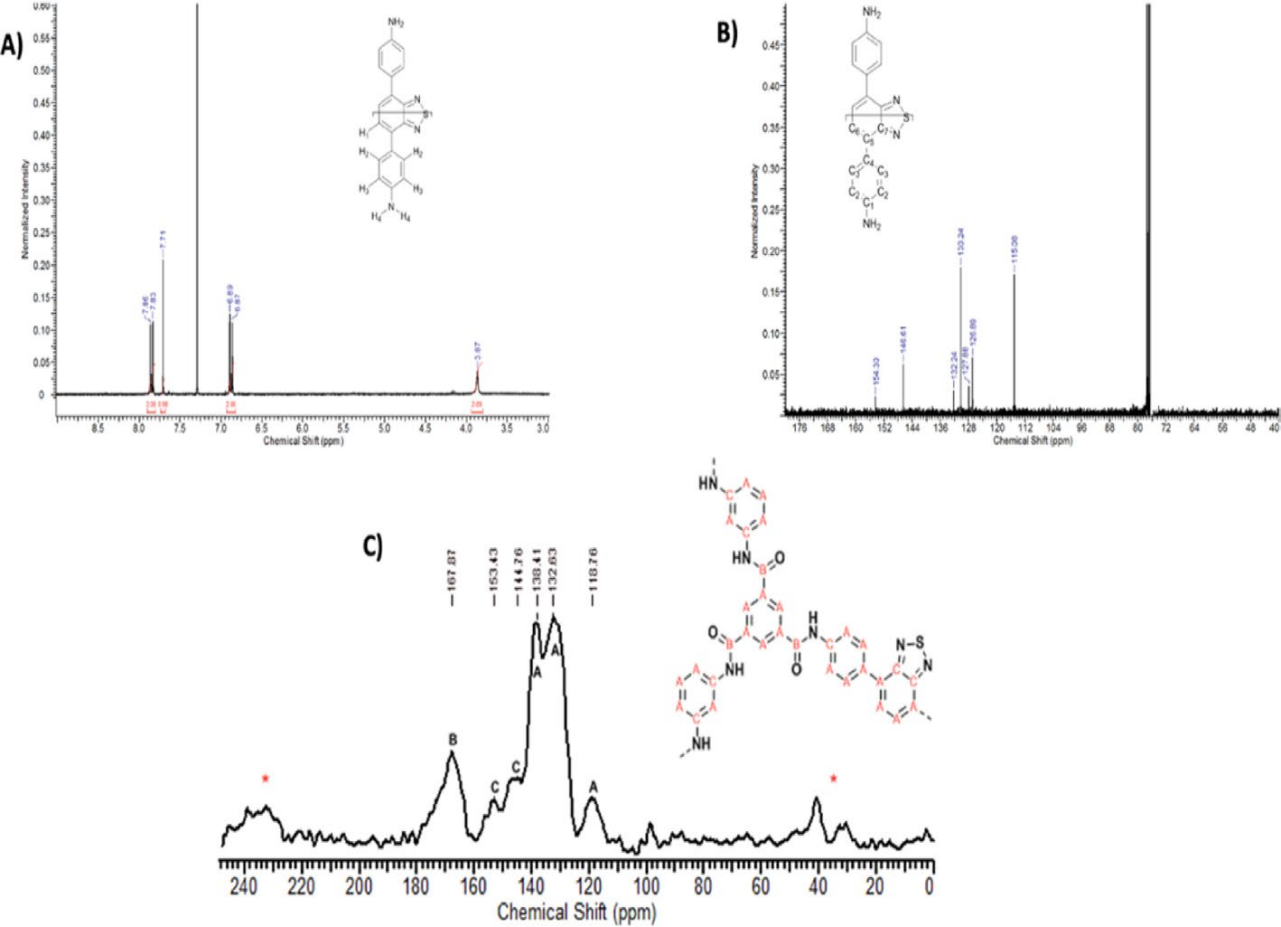
NMR spectra of ABT monomer and PA-ABT polymer. **A** ^1^H NMR of ABT monomer (300 MHz, 30 °C, CDCl_3_) δ = 3.87 (s, 2 H, H_4_), 6.88 (d, 2 H, H_3_), 7.71 (s, 1 H, H_1_), 7.84 (d, 2 H, H_2_). **B** ^13^C NMR of ABT monomer (400 MHz, 30 °C, CDCl_3_) δ = 115.08 (C_1_), 126.69 (C_7_), 127.68 (C_6_) 130.24 (C_2_), 132.24 (C_3_), 146.61 (C_4_), 154.30 (C_5_). **C** Solid state ^13^C NMR of PA-ABT (25%) (δ167.8 (O = C–N), 153.4, 144.7 (N–C), 138.4, 132.6, 118.7 (other aromatic–C). Peaks denoted by (*) indicate the presence of side bands

1 H NMR and 13 C NMR spectra of ABT monomer and solid state 13 C NMR of PA-ABT 25% are shown in Fig. 4A, B and C.

##  Experimental results

###  Initial testing of polymer and light exposure setup

**Fig. 5:**
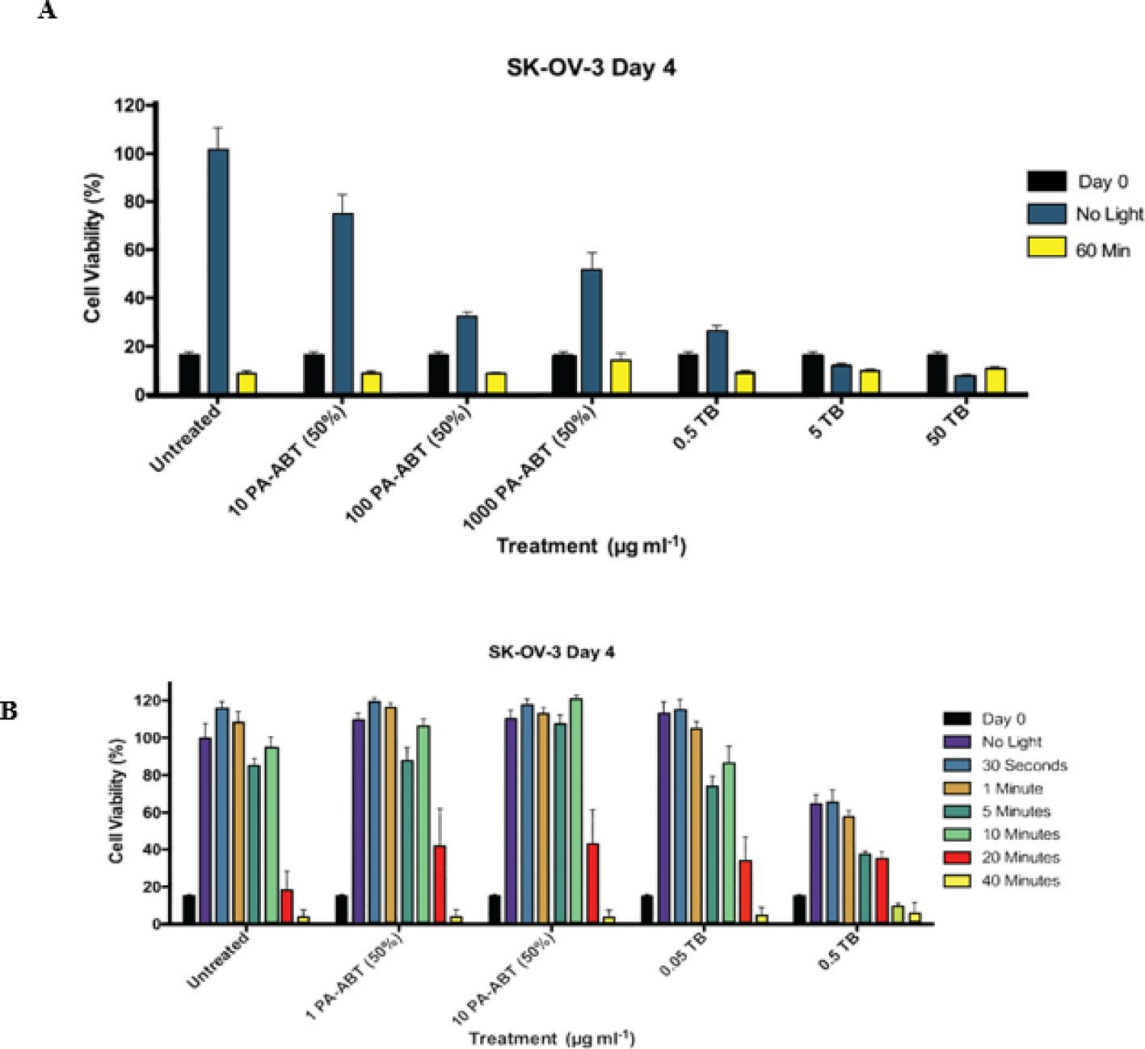
**A** Graph showing normalized reduction in cell number (100% determined by the untreated, no light control) of SK-OV-3 cells at day 0 (for comparison) and at day 4 following treatment. Cells were exposed to either no Light or 60 min of Light irradiation. A range of PA-ABT 50% polymer concentrations (10, 100 and 1000 µg.ml^−1^) and Toluidine Blue (TB) (0.5, 5 and 50 µg.ml^−1^) as a positive control alongside untreated cells as controls were used. Untreated *n* = 24, PA-ABT and TB treatments *n* = 6 at all concentrations and light exposures. **B** Graph showing normalised reduction in cell number 100% determined by the untreated, no light control) of SK-OV-3 cells at day 0 (for comparison) and at day 4 following treatment. Cells were exposed to no Light or to 30 s, 1, 5, 10, 20–40 min of Light. Treatments of PA-ABT 50% at 1 and 10 µg.ml^−1^ concentrations and Toluidine Blue (TB) at 0.05 and 0.5 µg ml^−1^ alongside untreated controls are shown. *n* = 12 at all treatments and light exposures

A series of initial optimization experiments were designed and carried out to find effective concentrations of polymer and optimum time lengths of Light irradiation for activation. Initially, PA- ABT 50% polymer was tested at 3 different concentrations; 10, 100 and 1000 µg.ml^−1^ and within each tested concentration the polymer was exposed to either no Light or 60 min of light irradiation **(**Fig. 5A**)**. Toluidine Blue (TB) was used as a potential positive control at concentrations of 0.5, 5 and 50 µg.ml^−1^. After 60 min of Light irradiation, a substantial reduction in cell number was observed. Similar observations were detected across the range of polymer concentrations. All concentrations of PA-ABT 50% caused a significant decrease in cell number with no Light exposure compared to untreated wells, with 100 µg.ml^−1^ having the greatest effect **(**Fig. 5A**)**. As a positive control, TB proved to be very effective at all concentrations in the presence or absence of light compared to the untreated cells. **(**Fig. 5A**)**.

After 60 min of light irradiation, a significant and large increase in cell number was obtained **(**Fig. 5A**)**, in order to overcome this problem, we tested shorter light exposure times (ranging from 30 s to 40 min) and lower concentrations of the photosensitizers PA-ABT (50% concentration) (1 and 10 µg.ml^−1^) and TB (0.05 and 0.5 µg.ml^−1^) to avoid excessive growth inhibition. The rationale behind this adjustment is that higher concentrations of the PA-ABT and TB were already effective even without light exposure, so using lower concentrations would help balance the treatment and prevent unnecessary damage caused by prolonged light exposure **(**Fig. 5B**)**. Increased Light exposure leads to a significant decrease in cell number, even in untreated cells, at 5, 20, and 40 min. Whilst the pattern of increasing Light exposure on cell number is similar between all treatments. Moreover, 10 µg.ml^−1^ PA-ABT 50% can be seen to significantly decrease cell number when compared to untreated cells at 5, 10 and 20 min of Light exposure. This effect is also seen in the 1 µg.ml^−1^ concentration at 20 min. TB behaves similarly to control cells at 0.05 µg.ml^−1^ but has significant impact on cell deathat 0.5 µg.ml^−1^.

###  PA-ABT (50%) causes growth Inhibition of SK-OV-3, PC-3 and T24/83 cells with an increased effect in combination with light in PC-3

**Fig. 6:**
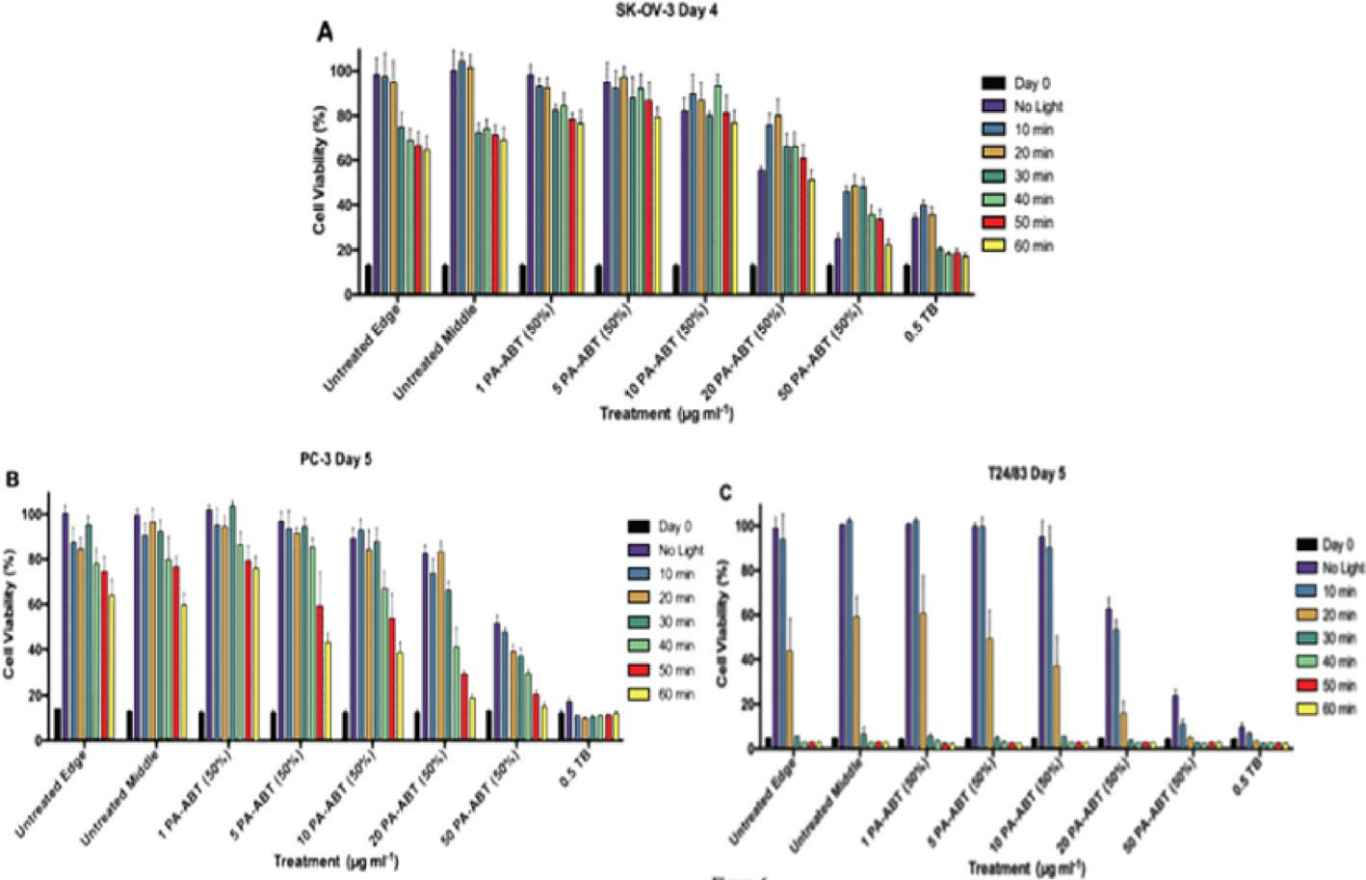
Graphs showing normalised reduction in cell growth (100% determined by the highest untreated, no light control) of **A** SK-OV-3 cells at day 4, **B** PC-3 cells on day 5 and **C** T24/83 cells at day 5 following treatment, day 0 shown for comparison. Cells were exposed to either no Light or 10, 20, 30, 40, 50–60 min of Light and treated with PA-ABT 50% at 1, 5, 10, 20 and 50 µg.ml^−1^ concentrations. Toluidine Blue (TB) at 0.5 µg.ml^−1^ was used as positive control. Untreated edge *n* = 18, all other treatments *n* = 6 at all light exposures

A range of PA-ABT 50% concentrations; 1, 5, 10, 20 and 50 µg.ml^−1^ and TB at 0.5 µg.ml^−1^ was used as a positive control were tested from none to 60 min of Light exposure in 10-minute intervals in SK-OV-3 **(**Fig. 6A**)**, PC-3 **(**Fig. 6B**)** and T24/83 **(**Fig. 6C**)** lines. SK-OV-3 were fixed on day 4, with PC-3 and T24/83 fixed on day 5.

In SK-OV-3 **(**Fig. 6A**)** both untreated controls showed substantial increased cell number starting from 30 + minutes of Light exposure. When cells treated with 5 and 10 µg.ml^−1^ of PA-ABT polymer concentrations, a significant decrease in cell number at Light exposures of 30 to 60 min was obtained when compared to untreated controls. At 20 µg.ml^−1^ a significant decrease in cell number was seen only in no Light, 10, 20 and 60-minutes exposure vs. untreated controls. Interestingly, at this concentration, a significant decrease in cell number at 10, 20 and 30-minutes exposure was obtained when compared to no Light exposure, this may suggest that Light exposure reduces the potency of PA-ABT polymer. When the polymer concentration increased to 50 µg.ml^−1^ a large decrease in cell proliferation was noticed across the range of Light exposures. In parallel to 20 µg.ml^−1^, the viability appeared to be significantly increased when exposed to 10, 20 and 30 min of light when compared to none.

In PC-3 **(**Fig. 6B**)** untreated cells suffer a slow increase in cell damage with increasing exposure to Light, with significant drops starting from 40 min. In no Light condition, only 20 and 50 µg.ml^−1^ concentrations of PA-ABT 50% showed a notable increase in cell damage. Interactions with Light can be seen at lower concentrations of polymer when comparing them to untreated controls at the same Light exposure. In this cell line, a significant increase in cell damage was detected at 30 and 40 min of Light exposure when cells treated with 20 µg.ml^−1^ of PA-ABT polymer; however, at 50 and 60 min of Light exposure, 5, 10 and 20 µg.ml^−1^ treated groups appeared to be significantly reduced. PA-ABT 50% at 50 µg.ml^−1^ show high inhibition across all the range of light exposures, but combinatorial effects are hard to see.

In T24/84 **(**Fig. 6C**)** and with 20 min, a significant and large decrease in cell growth of untreated cells is seen, with a potential reduction at 30 + minutes, showing extreme sensitivity to the Light treatment. Until the 20 µg.ml^−1^ PA-ABT 50% concentration, a significant decrease in cell growth was detected with a further drop at 50 µg ml^−1^.

###  PA-ABT (25%) displays low potency whilst 50% and 75% variants display significant growth inhibiting effects, increased by combination with light

At this point, 1 and 5 µg.ml^−1^ concentrations of PA-ABT 50% were excluded from further experiments due to low potency. However, 10, 20 and 50 µg.ml^−1^ concentrations PA-ABT 50%, alongside, 10 and 20 µg.ml^−1^ concentrations of PA-ABT 25% and 75% were tested.

**Fig. 7:**
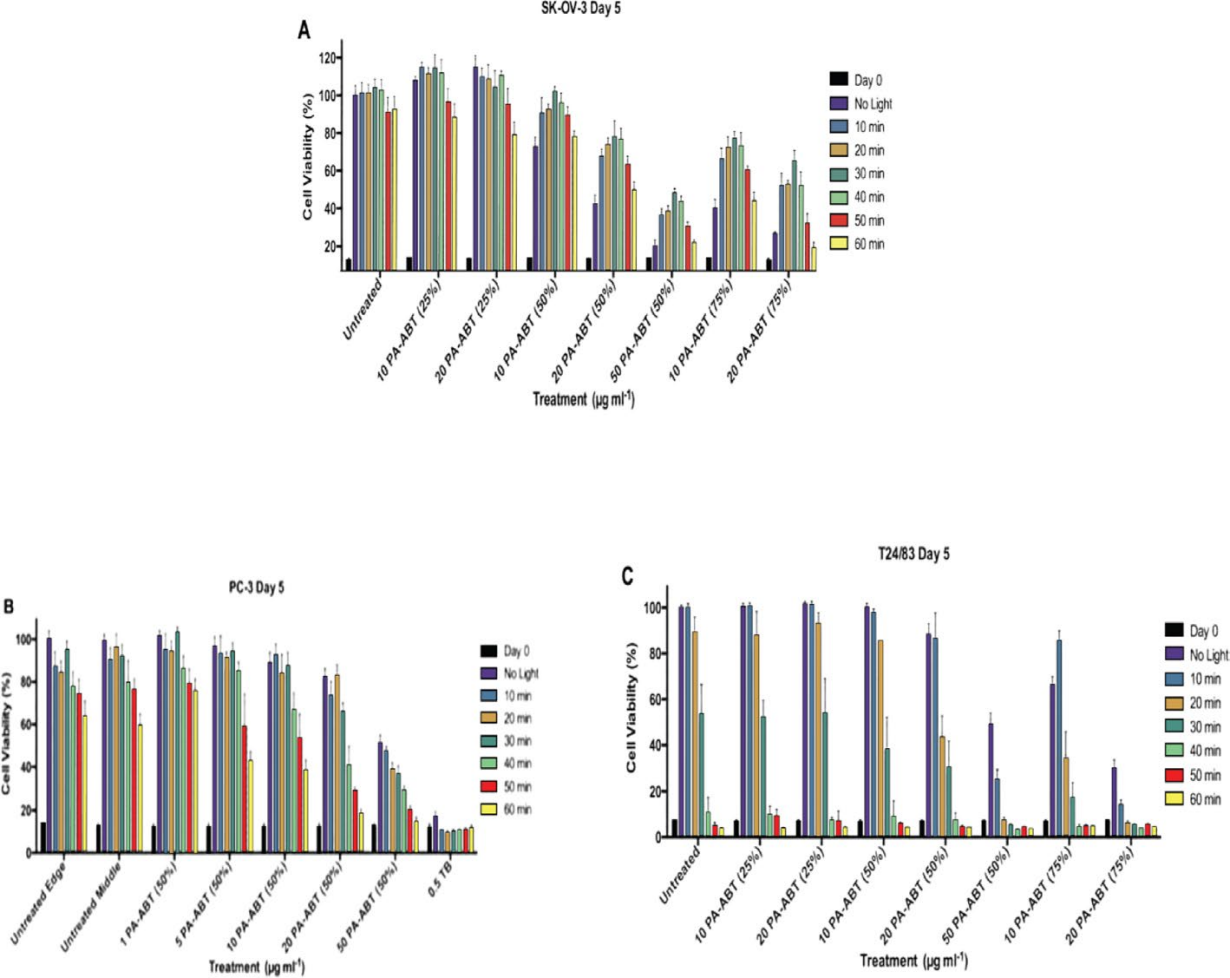
Graphs showing normalised cell growth inhibition (100% determined by the untreated, no light control) of **A** SK-OV-3 cells, **B** PC-3 cells and **C** T24/83 cells at day 5 following treatment, day 0 shown for comparison. Cells were exposed to either no Light or 10, 20, 30, 40, 50–60 min of light. Untreated edge and middle are shown to highlight differences in cell growth inhibition at various light exposures between the middle and edge of the plate, now not significant. Treatments of PA-ABT (25%) at 10 and 20 µg.ml^−1^, PA-ABT (50%) at 10, 20 and 50 µg.ml^−1^ and PA-ABT (75%) at 10 and 20 µg.ml^−1^ concentrations are shown. Untreated *n* = 18, all other treatments *n* = 6 at all light exposures

In SK-OV-3 **(**Fig. 7A**)** a significant increase in cell reduction number in untreated controls is only seen at 50 min of Light exposure, showing much less sensitivity compared to what was found previously. When cells were not exposed to Light and when treated with 10 µg.ml^−1^ of 25% PA-ABT, no significant difference was shown, whilst a dramatic reduction in cell growth was observed at 20 µg.ml^−1^ of PA-ABT when compared to control. At 10 minutes’ exposure, both concentrations showed increased cellular growth over controls, continuing at 20, 30 and 40 min for 10 µg.ml^−1^ and at 40 min for 20 µg.ml^−1^. It is not until 60 minutes’ exposure that 20 µg.ml^−1^ displayed a significantly increased reduction in cell growth, showing potential interaction with Light. PA-ABT 50% causes significantly increased cell damage, increasing with each concentration when compared with controls. However, Light exposure reduces this effect and decreases cell damage. At 10 and 20 µg.ml^−1^, 10–50 min of Light exposure results in significantly increased cell survival vs. none, whilst at 50 µg.ml^−1^ 10–40 min of Light irradiation produces this effect, showing negative synergy between Light and polymer. PA-ABT 75% behaves similarly to 50%, whilst being significantly more effective at increasing cell damage across the range of Light exposures when compared to the respective 50% concentrations. This is most notable in the non-light treated cells. Again, at 10 µg.ml^−1^ cell growth is increased from 10 to 50 minutes’ Light exposure vs. none with the same effect from 10 to 40 min at 20 µg.ml^−1^.

In PC-3 **(**Fig. 7B**)** a significant, but small reduction in cell damage is seen at 20–60 minutes’ Light irradiation compared to no Light exposure in controls. Promising data showed a significant and large increase in cell death vs. control at 60 min exposure and when cells treated with 50 µg.ml^−1^ 50% and 20 µg.ml^−1^ 75%; however, with no Light and 10 minutes’ exposure, there is no significant reduction in death with any concentration or variant of polymer. This indicates that at higher concentrations, light has a synergistic effect with PA-ABT, increasing its potency.

T24/83 **(**Fig. 7C**)** again displays high sensitivity to Light. A small but significant increase in deathis seen at 20 minutes’ exposure in untreated cells, with larger increases at 30 and 40 min. PA-ABT 50% polymer at both concentrations 20 and 50 µg.ml^−1^ result in significantly increased deathwith no Light exposure; however, 10 minutes’ Light exposure causes a significant increase at 50 µg.ml^−1^, showing interaction. This interaction also occurs at 20 minutes’ irradiation in 10, 20 and 50 µg.ml^−1^ concentrations, with significant decreases seen from no Light or 10 minutes’ exposure. These cells display significantly higher reduction in cell number than respective untreated exposures, showing the increase is not due to light alone.

Both 10 and 20 µg.ml^−1^ 75% concentrations led to significant increases in cell damage in no Light vs. controls. Oddly, 10 minutes’ exposure causes a significant decrease in cell damage from no Light at the 10 µg.ml^−1^ concentration, an interaction seen in SK-OV- 3 previously. In contrast, at 20 µg. ml^−1^, 10 minutes’ exposure further increased cell damage, showing interaction. 20 minutes’ irradiation results in 10 µg.ml^−1^ treated cell growth dropping significantly lower than in no Light, or compared to controls, whilst 20 µg ml^−1^ further reduces growth.

###  BTP-1/BTP-2 polymers

The PA-ABT polymers have been shown to be sucessful PS, as a result two new polymers **(**Fig. 2B and C) were tested to examine their potential enhanced photosensitizer activities.

**Fig. 8:**
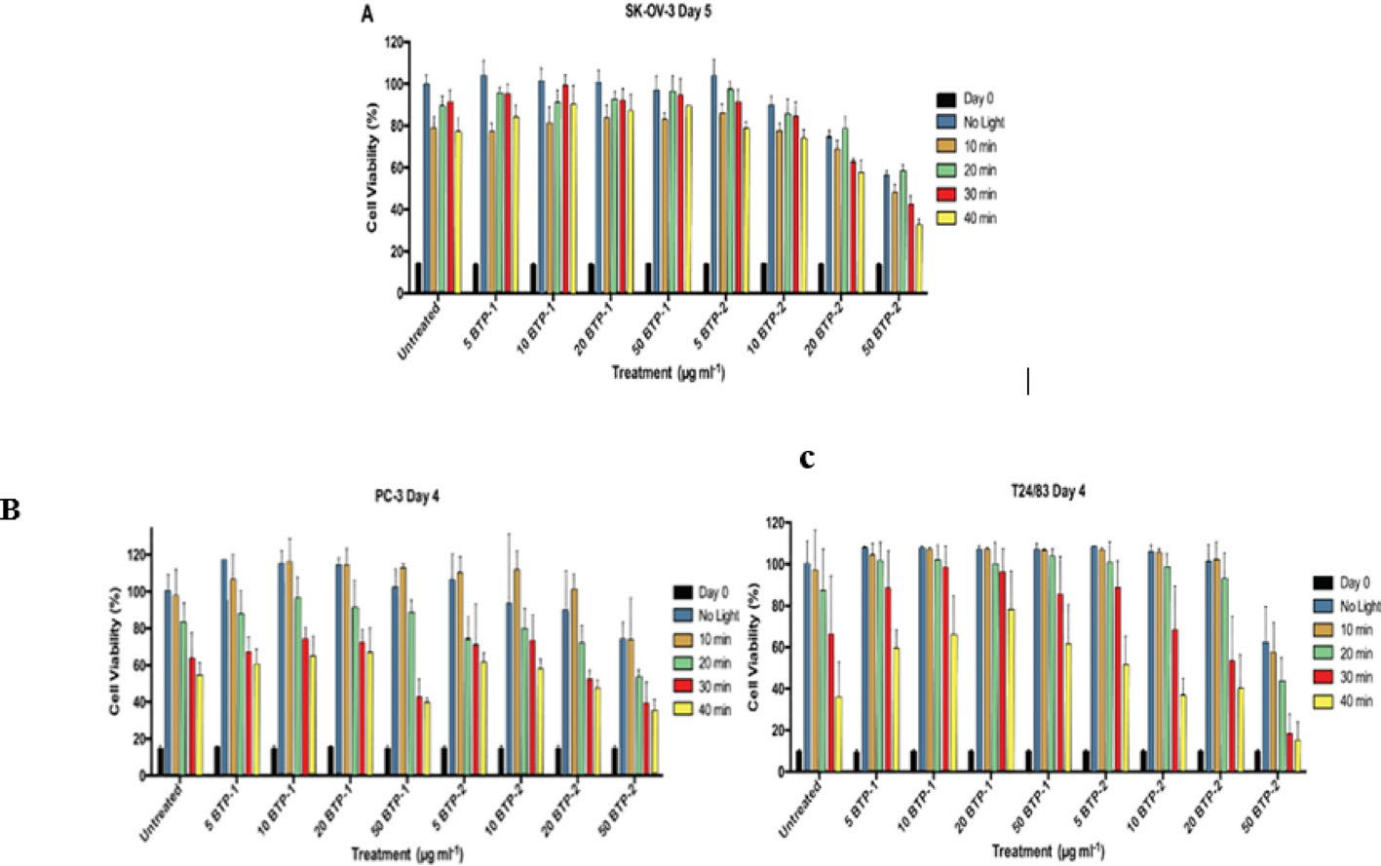
Graphs showing normalised cell growth inhibition (100% determined by the untreated, no light control) of **A** SK-OV-3 cells, **B** PC-3 cells and **C** T24/83 cells at day 5 following treatments, day 0 shown for comparison. Cells were exposed to either no Light or 10, 20, 30, or 40 min of light. Treatments of BTP-1 polymer at 5, 10, 20 and 50 µg.ml^−1^ and BTP-2 polymer at 5, 10, 20 and 50 µg. ml^−1^ concentrations are shown. Untreated *n* = 12, all other treatments *n* = 6 at all light expourse

In SK-OV-3, Light causes a significant increase in cell damage was observed at 10, 20, 30 and 40 min of exposure **(**Fig. 8A**)**. Decreased cell damage was displayed between untreated controls and cells treated with 10 µg ml^−1^ BTP-1 at 40 min of Light exposure. When cells were treated with 20 and 50 µg ml^−1^ BTP-2 polymer a significant increase in in cell death was seen with no Light in comparison to controls. Comparing to the untreated groups with increasing concentrations, a slight interaction is seen at 30 min in the 20 µg.ml^−1^ group and at 30 and 40 min at 50 µg.ml^−1^, with cell death increasing more linearly in the upper concentrations of BTP-2.

In contrast to SK-OV-3, PC-3 **(**Fig. 8B**)** showed greater sensitivity with significant and large increases in cell damage at 30 and 40 min vs. no light exposure in untreated controls. Like SK-OV-3, no significant difference in cell growth inhibtion was found at any concentration of BTP-1 compared to controls.

Comparing to BTP-1, BTP-2 only causes a significant increase in cell damage at 50 µg.ml^−1^ with no light exposure. A notable significant increase in cell growth inhibition caused by BTP-2 was shown at 50 µg.ml^−1^ with 20 minutes’ light exposure.

T24/83 cells **(**Fig. 8C**)** were affected by light in a similar manner to PC-3, with a significant and large increase in cell damage of untreated cells at 30 and 40 min of Light exposure. At 30- and 40-minutes’ exposure, only 10 µg.ml^−1^ and 20 µg.ml^−1^ of BTP-1 cause a potential decrease in cell death compared to their respective untreated controls. At various time points, BTP-2 polymer at 50 µg.ml^−1^caused a significant increase in cell damage vs. controls.

##  Conclusion

In this study, we investigated the photodynamic effect of PA-ABT and other related benzothiadiazole-based polymers on three human cancer cell lines in in vitro model. Our findings suggest that PA-ABT can reduce cellular growth in a dose dependent manner particularly at higher concentrations and under light exposure specifically in PC-3 and T24/83 cells. In T24/83 and PC-3 cells the polymer appeared to have no effect with no, or minimal Light exposure at lower concentrations, but following 30–60 minutes’ light irradiation displayed inhibitory effects compared to controls. BTP-1 and BTP-2 did not exhibit photodynamic activity under the tested conditions, with BTP-1 showed minimal efficacy on reducing cell growth even in combination with light and BTP-2 showing toxicity in darkness and inhibitory effects at higher concentrations but displayed little/no interaction with Light possibly due to solubility or delivery Limitations. Accordingly, additional studies should be performed using higher concentrations and optimized exposure parameters to accurately determine their potential effects. 60 min of Light irradiation presented a substantial decrease in untreated cell growth. This was the same across the range of polymer concentrations, with no significant difference between any at 60 min light exposure. All concentrations of PA-ABT (50%) appeared to increase cell damage with no Light exposure compared to untreated wells, with 100 µg.ml^−1^ having the greatest effect. The preliminary findings presented here indicate that benzothiadiazole-based polymers display growth-inhibiting effects in some human cancer cell lines, with differing levels of sensitivity observed suggesting cell-type-specific responses that require further investigation. The initial experiments investigated how light exposure influences cell survival, revealing a notable impact on all three untreated cell lines, with untreated SK-OV-3 cells considerably inhibited by 10 + minutes of light irradiation **(**Fig. 5A and B) and supplementary (Figure S2). Lining the box with light-absorbent black card significantly reduced this effect **(**Figs. 6A, 7A and 8A and Figure S3) suggesting that the previously reflective lining was leading to an increased light dose. Despite this, the light still had a notable impact at higher exposure times in SK-OV-3 and PC-3 whilst causing growth inhibition of T24/83 cells at exposures over 20 min. At 420 nm, the Light had a profound effect on cell growth in the visible range. However, this observation was noticed previously in studies that have shown that near-UV Light can cause DNA damage up to 434 nm in human cells [25] and up to 450 nm in Chinese hamster ovary cells [26] caused by endogenous photosensitizers leading to oxidative damage. Endogenous photosensitizers can enhance the efficacy of PDT by facilitating ROS production, endogenous photosensitizers occur naturally within cells, in conjunction with external light, these molecules can absorb light and allow the transfer of energy to oxygen to generate ROS mainly ^1^O_2_ and subsequent cellular responses [27]. These photosensitizers play vital roles in different biological mechanisms such as in photomedicine and trigger oxidative stress response within the cell, which induce cell death, including apoptosis and necrosis [28]. 

In several cases, PA-ABT increased growth inhibition with increasing light irradiation more so than independently. It was first noted in the PC-3 cell line **(**Fig. 6B**)** where concentrations of PA-ABT (50%) ranging between 1 and 20 µg.ml^−1^ were non-toxic in no Light, also in 10 and 20 min of Light. However, 30 to 60 min of light irradiation led to a notable increase in cell death when treated with different concentrations of PA-ABT **(**Fig. 7B**)**. Promising results were also found in T24/83 **(**Fig. 7C**)** with 10 and 20 µg.ml^−1^ PA-ABT (50%) showing little/no reduction in cell growth inhibtion with no or 10 minutes’ Light exposure. However, with 20 min Light these concentrations appeared to reduced viability vs. controls whilst 50 µg.ml^−1^ PA-ABT (50%) led to a notable inhibition of growth with no Light, the drops at 10 and 20 min of Light is more than that seen in untreated controls, which can be due to an interactive effect. The same is true of 20 µg ml^−1^ PA-ABT (75%) with 10 minutes’ exposure. In SK-OV-3, there was little beneficial interaction seen between the polymer and light but a notable drop in cell growth can be seen in **(**Fig. 7A**)**, with 20 µg.ml^−1^ PA-ABT (25%) at 60 minutes’ exposure compared to controls. These results suggest that the inhibitory potential of PA-ABT may be controllable by light exposure, potentially through singlet oxygen production. From minimal cytotoxicity in darkness and at lower concentrations to more effective reductions in cell viability at longer light exposure, though further investigation is required to confirm this mechanism.

Interestingly, unexpected decrease in cell damage was noticed in some cases when cells are treated with lower PA-ABT concentrations under light exposure, particularly in SK-OV-3 cells **(**Fig. 6A**)**, with 5 and 10 µg.ml^−1^ PA-ABT (50%) leading to increased cell survival at exposures of 30–60 min. The same effect can be seen in **(**Fig. 7A**)** with 10 µg.ml^−1^ PA-ABT (25%). The effect is more widely seen in PC-3 **(**Fig. 7B**)** where there is a smaller increase affecting all concentrations of each PA-ABT variant from 20 to 40 min, besides 10 µg.ml^−1^, 75%. While the mechanism behind this phenomenon remains unclear, it may involve photobleaching or may be the fact that insufficient singlet oxygen being produced by the polymer to cause significant damage to the cells, whilst still absorbing a great portion of incident light, thereby protecting the cells from photodecomposition. These findings emphasize the complexity of the photodynamic response and the need for further investigation.

Following introduction of the blacked-out box, all 3 SK-OV-3 experiments **(**Figs. 6A and 7A and Figure S3) showed an interesting interaction whereby cellular growth increased in PA-ABT treated cells exposed to Light, when compared to treated cells which are not. This is seen at several concentrations and exposures but only in cases where treatment produces inhibition with no Light. Results showed the inhibitory effect of 50 µg.ml^−1^ PA-ABT (50%) to be reduced at 20 and 30 min at height 1 (Figure S3). At 10, 20 and 30 min of Light irradiation there is a notable decrease in cell death in cells treated with 20 and 50 µg.ml^−1^ PA-ABT (50%) vs. no light **(**Figs. 6A and 7A**)** most profoundly shows this with all concentrations of PA-ABT 50% and 75% displaying increased cel growth from 10 to 50 min. The only example of this in another cell line is T24/83 **(**Fig. 7C**)** where the effect is seen only at 10 µg.ml^−1^ PA-ABT (75%) between no Light and 10 min. This observation of negatively affected cell death in light-exposed, PA-ABT-treated cells was consistently noted in one cell line, and the underlying mechanism remains unclear and is subjected to further investigations. Effects of light on the cells can be discounted, as there were no observations of light exposure improving cell growth in untreated cells. Instead, we believe that this may appear to be due a photobleaching effect, with light causing an inactivation of the polymer. It could be possible that cell growth increased due to a decreased singlet oxygen production, or conversely, that increased singlet oxygen production somehow leads to increased growth. Either way, it is not possible to explain these findings from these results, however, further experiments investigating the singlet oxygen levels may shed more light on this.

BTP-1 and BTP-2 did not show any effective photosensitizing activity under the conditions tested, and the data generated from these two polymers does not offer the depth of findings as seen in PA-ABT. BTP-1 showed no observable effect at the tested concentrations; however, this may be due to limited polymer availability within the wells, possibly due to poor solubility or dispersion issues. BTP-2 did show inhibitory effects at higher concentrations; however, these effects did not appear to be enhanced by light exposure, and the polymer demonstrated cytotoxicity in darkness. These findings may suggest that BTP-2 may act through a light-independent mechanism. Further experiments are needed to explore whether adjusting concentration or light exposure parameters could reveal photosensitizing activity, particularly for BTP-1, which showed no measurable effects under the current conditions. Testing higher concentrations or optimizing polymer delivery may help clarify their potential roles in light-activated therapies.

PA-ABT shows promise as a potential candidate in PDT treatment modality with light-modulated inhibitory effects observed in the studied cancer cell lines. However, varied responses across the cell lines may prove it more effective in specific cancers, stressing the need of further studies and investigations to fully understand its mechanisms and efficacy across different tumor types.

##  Discussion

In this study several important key aspects should be discussed in the context of PDT for bladder cancer treatment, mainly regarding the use of 420 nm Light wavelength. The use of an appropriate wavelength in PDT is critical for the efficiency of the treatment modality as it is affected by several important factors, such as, the penetration depth, Light scattering behaviour and the light- tissue interactions. Using shorter wavelength like 420 nm causes light scattering effect as light passes through the tissue, which reduces light penetration compared to longer wavelengths that are more effective for deep-seated tumours [29]. However, in some cases the use of such short wavelengths can be beneficial for superficial tumours, as in shallow tumours, the scattered light can still be able to activate the administrated photosensitizers. In terms of bladder cancer, when this type of cancer is diagnosed in its early, thin and superficial stages(e.g., stage 0 or stage 1 bladder cancer), the use of 420 nm Light becomes potentially more effective for these tumours as there is no requirement for deep penetration of Light, instead, the 420 nm light become more sufficient to activate the photosensitizers, since the tumour is located relatively close to the surface of the bladder lining, making shorter wavelengths more effective and non-invasive treatment in PDT modality [30]. Moreover, the shallow penetration of the 420 nm light can be advantages for cancer treatment in superficial, thin bladder cancer as it helps spare the normal underlying bladder tissue from being damaged by the photodynamic side effects. In this case the light targets primarily the superficial tumour’s surface where the PS accumulates, reducing or minimising the collateral damage to the healthy normal bladder tissues beneath the tumour [31]. On the other hand, when it comes to the thick and deep-seated bladder tumour, the shallow penetration become a problematic. Using such a shorter wavelength (420 nm) and due to the absorption and scattering of light when it travels through the cancerous tissue, it becomes weak or attenuated, this attenuation happens quickly, making any deeper tumour areas not affected by light even in early stages, because the deeply penetrated PS will not be effectively activated by the light, as a result the efficacy of PDT will potentially affected and it will decrease. In such cases, a longer wavelength of light that allows deeper penetration within the tumour tissue (such as 630–650 nm) would be more efficient and effective for successful PDT treatment [32]. Accordingly, while the use of shorter wavelengths Like 420 nm may limit depth efficacy, they can also reduce collateral damage to normal tissues, suggesting a need for a balanced approach in PDT applications for bladder cancer. For tumors with greater thickness, potential approaches to enhance light penetration can be used, such as the use of higher intensity light, longer wavelengths, or advanced delivery techniques (e.g., intratumoral injections or light delivery via endoscopic devices) that could help overcome these Limitations. However, in our study, it is important to stress that the synthesized PA-ABT polymer backbone can be changed to improve its performance, and the absorption wavelength can be adjusted or changed for better clinical performance. In this study, the Light dose was calculated and found to be 113 J/cm^2^ which is relevant with other studies. Previous studies, both in-vitro and clinical, have employed similar light doses in their investigations. Haddad et al. (1998) exposed B16 murine melanoma cells to photoradiation at doses of 50, 100, or 200 J/cm², which resulted in a significant increase in cell death [33]. In a clinical study, Usuda et al. (2018) administered PDT to seven patients, including five with adenocarcinoma and two with squamous cell carcinoma. The laser doses of 50 J/cm² and 100 J/cm² were determined to be feasible and effective, demonstrating that PDT at these light doses is a non-invasive and viable treatment option for peripheral early-stage lung cancer [34]. Furthermore, accurate measurement of the light dose can be done, and it is very critical for bladder illumination. The variation in the light dose can significantly affects the efficacy and toxicity of the PDT treatment. Several studies highlight the importance of precise dosimetry techniques including the use of fluorescence dosimetry, advanced irradiance monitoring systems and simulations to ensure uniform light distribution across the bladder wall [35, 36]. Also, other radiometric measurement can be applied using light sensors such as photodiodes and photomultiplier tubes [37]. This research study presents preliminary findings on a synthesized polymer designed for use in in vitro models. The primary objective of this study was to evaluate the efficacy of the synthesized polymer in in vitro models using various cancer cell lines. These models lack the complexity of the tumor microenvironment and are subject to oxygen limitations that can impact PDT efficiency when compared to clinical studies using real tissues. In this work, the SRB assay was employed to assess cell death, the SRB assay was selected as a quantitative and efficient alternative to measure cell viability and proliferation. It is widely recognized as a reliable method for evaluating cytotoxic effects in-vitro, which can be used as an assay that reflects indirectly cell number. Although SRB is used specifically to detect the cellular protein content but also can be used to assess cell proliferation, since cellular protein content correlates with cell mass, it is often used as a high-throughput method for screening and assessing cell cell growth and metabolic activity after certain treatments such as drug efficacy including those used in PDT offering a fast and reliable approach to complement more extensive assays like clonogenic assays. The SRB assay binds directly to protein in the cells, where the dye binds to the basic amino acids in proteins, and the intensity of the dye’s color is directly proportional to the amount of protein in the sample, which is often used as an indicator of cell density or death [38]. In terms of future experiments, more accurate cell viability assays such as colony formation assay (clonogenic assays) can be used to confirm the in-vitro findings. However, this assay is not suitable for determining therapeutic efficacy in vivo, where more comprehensive and clinically relevant assessments are required. In laboratory settings, the application of light in PDT typically occurs directly on cultured cancer cells or tissue samples. While these experiments allow for controlled conditions, they do not accurately replicate the complex process of light delivery to deep-seated solid tumors within the human body [39]. Even with the use of longer wavelengths, assessing the efficiency of light penetration and delivery remains challenging. This is particularly true when oxygen levels in the target tissue or cells are insufficient for ROS generation, or when the PS is not uniformly distributed within the tumor region [40]. Efficacy of PDT is primarily influenced by the suboptimal activation of the PS, which depends on several factors, including the concentration of the PS in the tumor, light penetration and delivery, and the availability of oxygen in the target area for ROS production [41]. While these factors can be controlled in in vitro models, it is important to note that such controlled conditions do not accurately reflect in vivo scenarios, where tumor heterogeneity and imperfect light penetration and delivery present significant challenges [42]. 

In conclusion, our findings indicate that this approach exhibits novel effects on cancer cells. In the future, it might be possible to integrate immobilized photosensitizers into membrane-like structures which can be combined with externally controllable light sources, which after extensive validation and testing might contribute to future localized therapeutic approaches for solid tumors.

##  Limitations of the study

Several Limitations should be acknowledged when interpreting the results of this study. While PA-ABT demonstrated promising characteristics as an ideal PS for PDT; however, the results generated from this study should be considered as preliminary data, as the presented data lack data analysis, therefore, all drawn conclusions are from preliminary data that need further investigations and analysis; however, we believe the presented results still provide valuable preliminary observations that could inform future studies. Moreover, the experiments are Limited to 2D in vitro cell culture models, which do not fully recapitulate the complexity of tumor microenvironments found in vivo. Additional studies will be conducted to elucidate the underlying mechanisms of action, optimization of polymer formation, and assess its efficiency in more relevant models such as three-dimensional tissue and in vivo systems that reflects the heterogeneous oxygenation environment and immune system interactions that can be found in the physiological environment of a tumour tissue. Second, while the light exposure settings were carefully controlled, the distribution and penetration of light in tissue-like environments were not assessed, which may affect the clinical translatability of these findings. Additionally, variability in response between cell lines highlights the need to better understand the mechanisms underlying differential sensitivity to PA-ABT and related polymers. The observed enhancement of cell growth at certain light doses and concentrations was unexpected and remains unexplained, requiring further mechanistic exploration. Bladder tumors begin as superficial lesions confined to the mucosa or submucosa, making them vital target to PDT. This was reported on one of the earliest clinical successes with PDT in the New England Journal of Medicine following treatment of bladder tumors in China, although the work primarily utilized hematoporphyrin derivative and red light, not UV irradiation [43].

However, and in contrast to the bladder cancer, ovarian and prostate cancers frequently present with deeper, more heterogeneous, or disseminated tumor masses. This structural difference presents a significant challenge for the penetration depth of light and the delivery of photosensitizers. Therefore, while PDT has demonstrated promise in treating early-stage bladder cancer due to its superficial nature, adaptations in photosensitizer design, light delivery systems, and potentially combinatorial therapies are essential for achieving therapeutic efficacy in less accessible tumors such as those found in the ovary or prostate.

Furthermore, only a limited range of polymer concentrations and light wavelengths were tested, which may not represent the full therapeutic window or optimal conditions for PDT efficacy. Finally, while initial characterization of the polymers was performed, comprehensive assessments of long-term stability, biodistribution, and degradation in biological systems were beyond the scope of this study and should be addressed in future work.

##  Future work

Future work will focus on in-vivo validation to further assess the polymer’s potential for clinical applications. While this study focused primarily on inhibitory effects in three cancer cell Lines which can be controlled by Light exposure. Furthermore, we aim to create a ground-breaking treatment in response to the unmet clinical need of bladder cancer through a radical, world first in PDT from an implantable medical microsystem. PDT has demonstrated potential in treating lung and skin cancers. However, it has not yet been widely applied using implantable therapies across any treatment approach due to several reasons including biological, clinical and technical limitations. Accordingly, these implantable LED based PDT devices represent a vital and promising future for selective, target and localized cancer treatment. Providing effective and potential applications for variety of deep-seated cancer types, including bladder cancer. By discovering the efficacy requirements of photodynamic polymers to the responses of 2D and 3D cancer cells, we will create an entirely new technology PDT via implantable microsystems. Although fibre optics have been a reliable method for light delivery in PDT, however, implantable wirelessly powered LED microsystem with targeted drug delivery systems hold significant potential in advancing PDT for bladder cancer, particularly for non-muscle-invasive bladder cancer (NMIBC) offering a promising alternative to traditional fibre-optic systems including, biocompatibility, flexibility, ease of design, improved light control, minimized tissue damage, and ease of use. Importantly, the LED microsystem is easier to integrate into implantable devices due to its miniaturisation (smaller size) and flexibility that facilitates the precise placement of the light source directly into the bladder wall. These devices are powered remotely through radiofrequency signals, which eliminates the need for external wires and for repeated intravesical instillations, therefore, enhancing the treatment efficacy for tumors in deep-seated lesions specifically within the bladder, providing a patient-friendly option in the treatment of bladder cancer.

Furthermore, these implantable LEDs can emit Light at shorter and longer specific wavelengths, such as 405 nm and 660 nm, which can activate a wide range of photomedicines. The device is designed to ensure a direct and specific penetration of light to the tumor site, increasing the efficacy of PDT with minimal damage to the surrounding healthy tissues.

We propose extending our research to address a radio micro-system that can provide wireless measurement readouts, this will be made up of an antenna-connected microcontroller device and an RFID chip. As a result, the transponder/sensor in our system will be implanted, wireless, and battery-free. The transponder load modulates a carrier frequency transmitted by the reader in this radio frequency identification system. An external reader that activates the transponder wirelessly and decodes the radio-frequency signals it emits.

##  Contribution of the study

This study has performed the initial assessment of 3 different benzothiadiazole-based polymers on cancer cell Lines in 2D culture. Treating cell Lines from 3 different tissues has shown promising results but also variability in sensitivity between cells. This suggests that as a possible PDT treatment, such polymers may only be effective in specific cancers. It is evident that further work is required to optimise administration and assess the cause of different responses between cell lines.

## Supplementary Information

Supplementary Material 1.

## Data Availability

Research Data Policy and Data Availability Statements Data available on request from the authors: “Data supporting this study are included within the article” The data that support the findings of this study are available from the corresponding author, [Rolan Mansour], upon reasonable request.

## Abbreviations

ABT: aminophenyl-substituted benzothiadiazole derivative
BTP: Benzothiadiazole-based conjugated polymer
ECACC: European collection of authenticated cell cultures
MPD: m-phenylendiamine
PA: Polyamide
PDT: Photodynamic therapy
^1^O_2_: Singlet oxygen
PSs: Photosensitizers
SRB: Sulforhodamine B
TB: Toluidine blue
TMC: trimesoylchloride
