# Supplementary material for: In vitro assessment of benzothiadiazole-based photoactive polymers against ovarian, prostate and bladder cancer cell lines for photodynamic therapy
Source: Discov Oncol. 2025 Oct 14;16:1863. doi: 10.1007/s12672-025-03654-1 (PMC12521685; doi:10.1007/s12672-025-03654-1)
Supplement: Supplementary file 1 — Supplementary Material 1. [file 12672_2025_3654_MOESM1_ESM.docx]

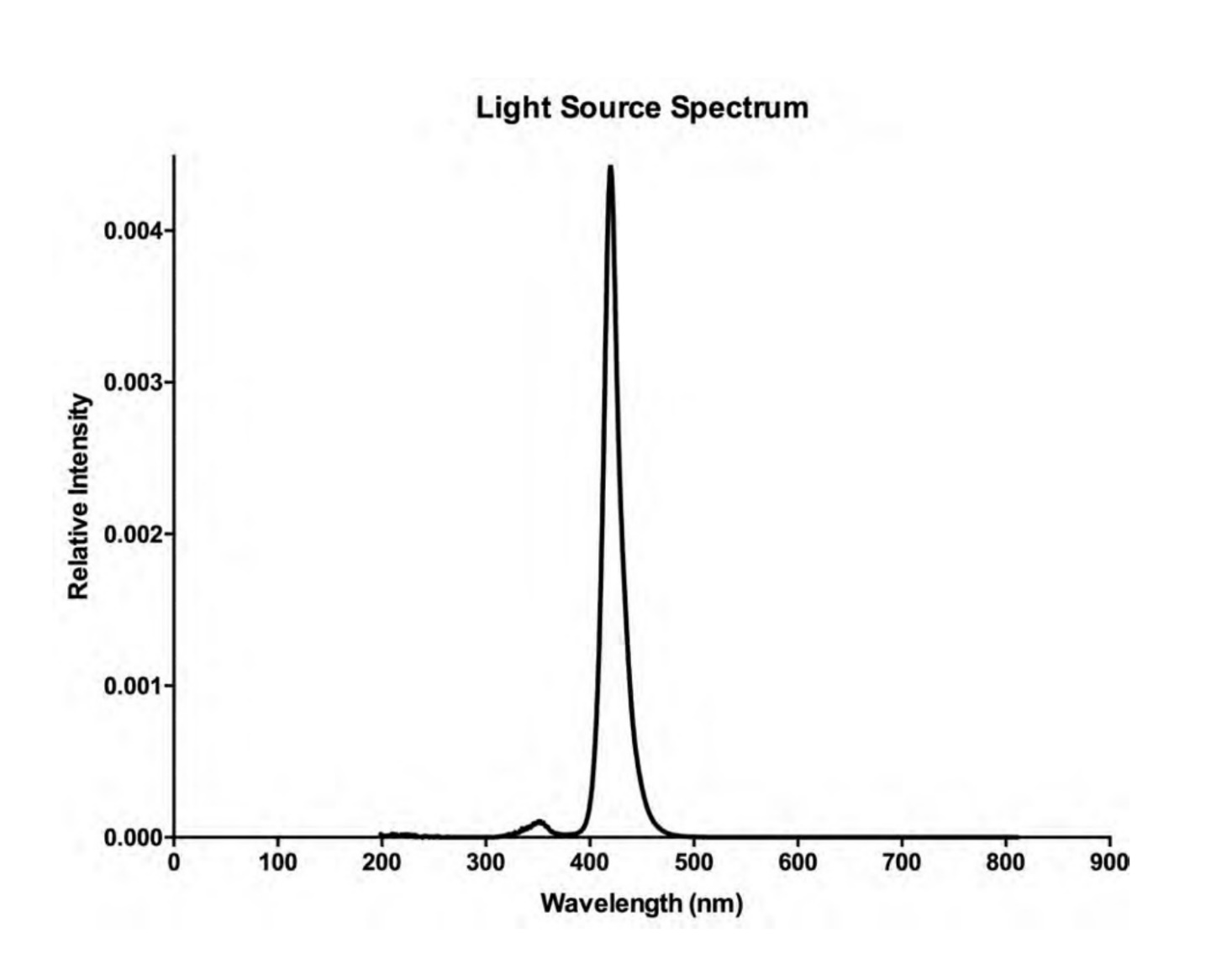


**Figure S1. Light source spectrum**. Spectrum showing relative intensity of wavelengths 199–811 nm output by the light source. Information obtained from 20 random LED chips and supplied by OSA Opto Light GmbH. Note that whilst the majority of outside is in the 410–440 nm range, a small peak is seen at 350nm.


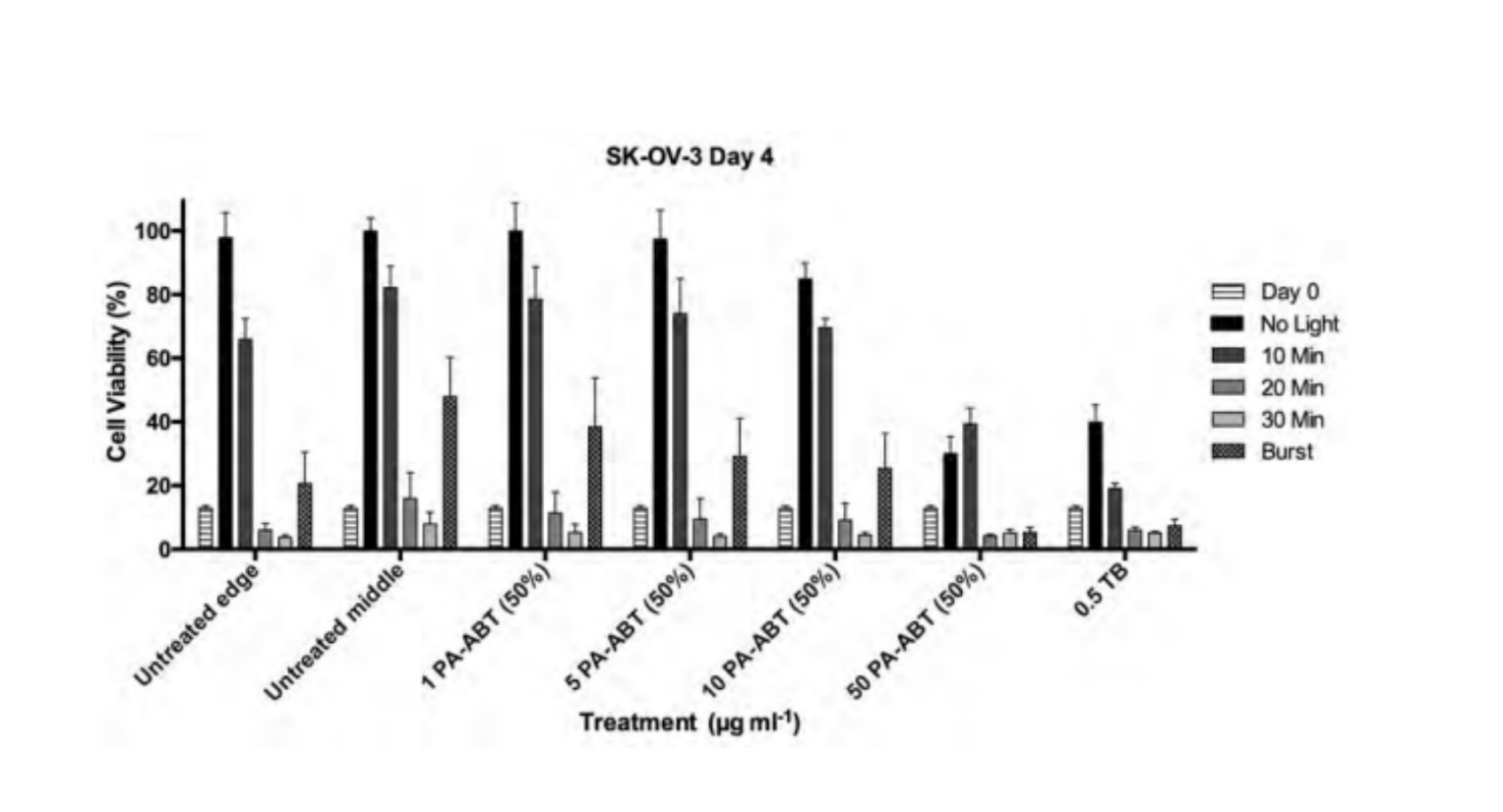


**Figure S2.** Graph showing normalised cell death (100% determined by the highest untreated, no light control - in this case, untreated middle) of SK-OV-3 cells at day 0 (for comparison) and at day 4 following treatment. Cells were exposed to either no light, 10 minutes, 20 minutes, 30 minutes or a 4x5 minute “burst” exposure of light. Untreated edge and middle are shown to highlight differences in cell growth at various light exposures between the middle and edge of the plate. Treatments of PA-ABT (50%) at 1, 5, 10 and 50µg ml^− 1^ concentrations and Toluidine Blue (TB) at 0.5µg ml^− 1^ are shown. Untreated edge n = 24, all other treatments n = 6 at all light exposures.

^
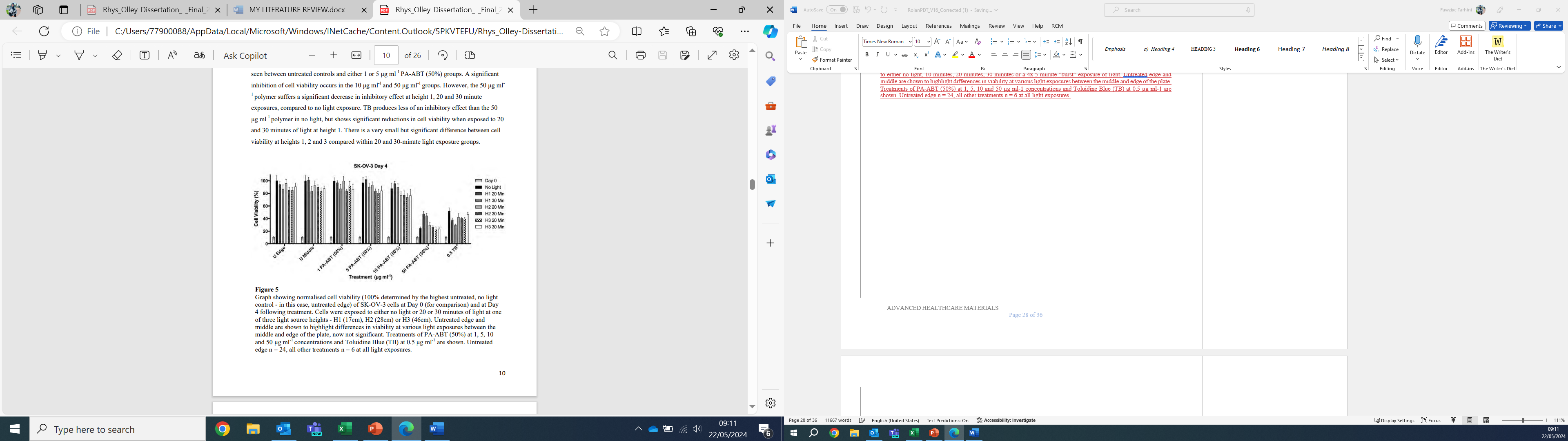
^

**Figure S3.** Light source height has minimal effect on cell death, lining box with black paper reduces spatial variation of light effect. Graph showing normalised cell damage (100% determined by the highest untreated, no light control - in this case, untreated edge) of SK-OV-3 cells at day 0 (for comparison) and at day 4 following treatment. Cells were exposed to either no light or 20 or 30 minutes of light at one of three light source heights - H1 (17cm), H2 (28cm) or H3 (46cm). Untreated edge and middle are shown to highlight differences in death at various light exposures between the middle and edge of the plate, now not significant. Treatments of PA-ABT (50%) at 1, 5, 10 and 50µg ml^− 1^ concentrations and Toluidine Blue (TB) at 0.5µg ml^− 1^ are shown. Untreated edge n = 24, all other treatments n = 6 at all light exposures.
